# Intrinsic and Extrinsic Aspects on *Campylobacter jejuni* Biofilms

**DOI:** 10.3389/fmicb.2017.01332

**Published:** 2017-07-18

**Authors:** Roberta T. Melo, Eliane P. Mendonça, Guilherme P. Monteiro, Mariana C. Siqueira, Clara B. Pereira, Phelipe A. B. M. Peres, Heriberto Fernandez, Daise A. Rossi

**Affiliations:** ^1^Laboratory of Applied Animal Biotechnology, Federal University of Uberlândia Uberlândia, Minas Gerais, Brazil; ^2^Laboratory of Molecular Epidemiology, Federal University of Uberlândia Uberlândia, Minas Gerais, Brazil; ^3^Institute of Clinical Microbiology, Universidad Austral de Chile Valdivia, Chile

**Keywords:** campylobacteriosis, poultry industries, chicken juice, capacity of biofilm formation, genetic apparatus, resistance to biocides

## Abstract

Biofilm represents a way of life that allows greater survival of microorganisms in hostile habitats. *Campylobacter jejuni* is able to form biofilms *in vitro* and on surfaces at several points in the poultry production chain. Genetic determinants related to their formation are expressed differently between strains and external conditions are decisive in this respect. Our approach combines phylogenetic analysis and the presence of seven specific genes linked to biofilm formation in association with traditional microbiology techniques, using Mueller Hinton and chicken juice as substrates in order to quantify, classify, determine the composition and morphology of the biomass of simple and mixed biofilms of 30 *C. jejuni* strains. It also evaluates the inhibition of its formation by biocides commonly used in industry and also by zinc oxide nanoparticles. Genetic analysis showed high heterogeneity with the identification of 23 pulsotypes. Despite the diversity, the presence of *flaA, cadF, luxS, dnaJ, htrA, cbrA*, and *sodB* genes in all strains shows the high potential for biofilm formation. This ability was only expressed in chicken juice, where they presented phenotype of a strong biofilm producer, with a mean count of 7.37 log CFU/mL and an ultrastructure characteristic of mature biofilm. The composition of simple and mixed biofilms was predominantly composed by proteins. The exceptions were found in mixed biofilms with *Pseudomonas aeruginosa*, which includes a carbohydrate-rich matrix, lower ability to sessile form in chicken juice and compact architecture of the biofilm, this aspects are intrinsic to this species. Hypochlorite, chlorhexidine, and peracetic acid were more effective in controlling viable cells of *C. jejuni* in biofilm, but the existence of tolerant strains indicates exposure to sublethal concentrations and development of adaptation mechanisms. This study shows that in chicken juice *C. jejuni* presents greater potential in producing mature biofilms.

## Introduction

*Campylobacter jejuni* is one of the pathogens most commonly involved in food-borne gastroenteritis worldwide. It infects about one million people in the United States each year and in Europe this rate reaches more than 200,000 (Scallan et al., 2011; European Food Safety Authority, 2015). In addition, an estimated number of 1/1,000 clinical cases may result in more severe neurological conditions, including Guillain-Barré Syndrome (Nachamkin et al., 1998).

The main reservoir of this microorganism is the intestinal tract of birds and other endothermic animals, and is often isolated in chicken meat. Generally, consumption of this undercooked meat is the cause of human host infection (Guyard-Nicodeme et al., 2013). The risk is consistent with the high levels of contamination found in studies conducted in Europe, USA and United Kingdom, which shows contamination higher than 70% in chicken carcass flocks (Batz et al., 2012; Lawes et al., 2012; European Food Safety Authority, 2015).

Due to the large number of reported cases of campylobacteriosis, it has become necessary to use epidemiological typing, method that allows the characterization and discrimination of bacterial strains. The data obtained in these investigations can be used by public health surveillance in identifying the causes of food outbreaks (Nakari, 2011). Among these methods, PFGE, pulsed-field gel electrophoresis, is considered the gold standard in bacterial epidemiological analyzes, since it allows a high discriminatory power compared to other techniques (Goering, 2010).

The paradox between the rigorous growth conditions of *C. jejuni* in the laboratory and the ubiquity as an effective and constant pathogen in chicken samples represents one of the most notable characteristics of *C. jejuni* (Mihaljevic et al., 2007). One of the strategies that *C. jejuni* can use to overcome its fragility in the face of environmental hostility is the ability to form biofilms. These structures represent a mode of growth and survival, in which the bacterial transits from free living to sessile form, attached to a biotic or abiotic surface surrounded by a viscous matrix that protects from stressful environmental conditions (Kostakioti et al., 2013). These communities increase the survival of this microorganism under unfavorable conditions, such as the presence of antibiotics and chemical agents (Trachoo and Frank, 2002; Joshua et al., 2006; Ica et al., 2012; Drozd et al., 2014).

A serious problem in the chicken processing industries is the insufficient removal of organic material composed of a complex mixture of carbohydrates, proteins, lipids, and sugars (Chmielewski and Frank, 2007) of the surfaces, which provides an ideal medium for microorganisms to multiply and survive. This environment assists in bacterial fixation to surfaces by altering the physicochemical properties of the surface and by the greater availability of nutrients (Dat et al., 2010; Hwang et al., 2012). Trying to simulate the nutritional conditions on the abiotic surfaces during processing, a model system with “chicken juice” (Brown et al., 2014) is used, based on the supplementation of culture medium with defrosted filter-sterilized poultry exudates (Birk et al., 2006).

The extracellular matrix is an essential component of bacterial biofilms, and normally, corresponds for more than 90% of the dry mass of a biofilm (Flemming and Wingender, 2010). In addition, it allows the cells to remain hydrated and metabolically active, imprisoning nutrients and liquids near the bacterial cells. It also reduces the access of large molecules, such as antimicrobials (Billings et al., 2013), allowing bacterial persistence, beyond being structurally important, once it maintains the biofilm shape and ensures its cohesion (Sutherland, 2001). Knowing the composition and architecture of the extracellular matrix of biofilms is important, as it helps in the use of tools that improve efficiency and disinfection strategies.

The molecular mechanisms that regulate biofilm formation of *C. jejuni* are still poorly understood. Some of the genes involved in the process include the ones responsible for cell motility (*flaA*) (Reuter et al., 2010), cell adhesion (*cadF*), quorum-sensing (*luxS*) (Plummer, 2012) and stress response (*dnaJ, cbrA, htrA*, and *sodB*) (Oh and Jeon, 2014).

The biofilm formation is flagella-mediated at the first moment of the adhesion, together with the proteins involved in cell adhesion, although its functionality is not crucial (Svensson et al., 2014). Detection of quorum-sensing markers indicates ability of binding between cells, development and detachment of biofilm (Plummer, 2012). Already the markers involved in the stress response play a decisive role, contributing to a superexpression of the capacity of formation of sessile cells (Oh and Jeon, 2014).

The aim of this study was to carry out a phylogenetic analysis on *C. jejuni* strains isolated from chicken carcasses destined for national market and also to exportation, followed by a qualitative and quantitative study on the formation of biofilms, including molecular aspects involving the presence of specific genes, the architecture and composition of these structures and also the interaction of these strains in mixed biofilms under conditions with and without supplementation with chicken juice. Finally, the objective was to evaluate the performance of different chemical agents in the removal of *C. jejuni* bacterial biomass to establish control strategies at industry.

## Materials and methods

### Strains and growth conditions

The study was conducted with 30 *C. jejuni* strains from the analysis of 280 cooled chicken carcasses ready for commercialization from September to November of 2015, from a Brazilian poultry exporting industry, with a complete production cycle.

Isolation was previously performed according to International Standards Organization (2006) and identification of the species was done by multiplex PCR according to Harmon et al. (1997). After confirmation, the strains were stored at −80°C in UHT skimmed milk.

To perform subcultures for reactivation, the strains were seeded for 48 h in Bolton broth (Oxoid) supplemented with 5% of defibrinated ram blood (Laborclin) at 37°C in microaerophilic conditions (Probac), followed by plaque peal in CCDA Agar (*Campylobacter* Blood-Free Selective Agar Base) (Oxoid) incubated under the same conditions (International Standards Organization, 2006).

In the adhesion and biofilm assays, the strains were cultured for 48 h under microaerophylia at 37°C in 20 mL of Mueller Hinton broth (MH) (Difco), using as inoculum the culture present in the plates of CCDA Agar. In parallel, these assays were performed using 20 mL of Mueller Hinton broth supplemented with 5% of chicken juice (Birk et al., 2004)—equivalent to the 100% concentration according to Brown et al. (2015)—to simulate the conditions of industry. After growing in both conditions, the suspensions were adjusted to an OD_600_ = 0.22 to 0.28, corresponding to a count of 10^4^ CFU/mL. The cells were centrifuged (5,000 rpm, 10 min, 4°C) and washed twice (0.9% NaCl) before the beginning of cultures for adhesion and biofilm assays.

The scanning electron microscopy (SEM) analysis was performed for simple (only *C. jejuni*) and mixed biofilms (*C. jejuni* paired with *Salmonella* sp., *Escherichia coli, Pseudomonas aeruginosa* or *Staphylococcus aureus*). The inoculum preparation, growth conditions and incubation were the same as previously described for *Campylobacter*: microaerophilic atmosphere at 37°C for 48 h. Both, in the SEM and in the biofilm matrix composition assay, only three *C. jejuni* strains, phylogenetically distinct by PFGE and with different classifications of BFI (Biofilm Formation Index) were used.

In phylogenetic analysis by PFGE, the bacteria were cultured at 42°C overnight in a brain and heart infusion agar (BHI agar) with 5% of defibrinated sheep blood under microaerophilic conditions. The present culture on the agar was resuspended in saline (0.85% NaCl) until reaching OD_610_ = 0.570–0.820 for carrying out the enzymatic digestion process.

Controls used in the study were *C. jejuni* strains (ATCC 33291, NCTC 11351, and IAL 2383) and *C. coli* (ATCC 43478). For mixed cultures, *Escherichia coli* strain (ATCC 25922), *P. aeruginosa* (PAO 1), *Salmonella* Enteritidis (ATCC 13076), and *Staphylococcus aureus* (ATCC 25923) were used.

### Adhesion test

The adhesion test was performed according to Sulaeman et al. (2009) with modifications. Briefly, 200 μl of the bacterial suspension containing 10^4^ cells prepared in MH broth and MH with 5% of chicken juice was added in 96-well plates. After incubation for 4 h at 37°C under microaerophylia, the adherent bacteria were washed twice with 0.9% NaCl solution and collected in wells filled with the same solution by scraping during 90 s. The obtained cell suspension was serially diluted and seeded in CCDA agar for enumeration in CFU. All strains were evaluated in triplicate and in three independent replicates.

### Qualitative biofilm formation test

Biofilms were formed as described by Kudirkiene et al. (2012), with modifications. Briefly, 200 μl of the bacterial suspension containing 10^4^ cells prepared in MH broth and MH with 5% of chicken juice was added in 96-well plates. For biomass formation, the plates were incubated for 48 h at 37°C in microaerophilic conditions.

After incubation, the media were removed, the wells were washed twice with 0.9% NaCl solution and dried for 30 min at 55°C. Total biomass was measured by fixation with 0.1% Crystal Violet (LaborClin) for 5 min, followed by elution with alcohol-acetone solution, containing 80% of ethanol and 20% of acetone (Synth®). The eluted dye was removed from each well and placed in a new 96-well microtiter plate for reading at OD_595_ (BA–bacteria adhered). The assays were done with eight replicates for each strain in three replicates. For the determination of the Biofilm Formation Index, the following formula was used:

$$\text{BFI}=\frac{\text{BA}-\text{PC}}{\text{BS}}$$

Where BFI represents the final result regarding the Biofilm Formation Index, BA the optical density obtained in the mixture of bacteria adhered, PC the absorbance value in the control wells without microorganisms, BS the optical density (OD_600_) of the suspended cultures in MH and MH with 5% of chicken juice (Naves et al., 2008). The final classification followed Table 1.

**Table 1:** Classification of biofilm formation index.

**Strong**	**Medium**	**Weak**	**Nonexistent**
≥1.10	0.70–1.10	0.35–0.69	<0.35

### Quantitative biofilm formation test

The number of sessile cells was determined by counting in CFU. After biofilm formation as described in the previous item, the wells were washed twice with a 0.9% NaCl solution, and the biomass was removed by scraping the wells for 90 s. The obtained cell suspension was serially diluted and plated on CCDA agar plates to obtain the number of CFU. All the assays were performed in triplicate, on three independent occasions.

### Identification of specific genes

The genomic DNA was extracted by the Wizard Genomic DNA Purification Kit (Promega), following the protocol provided by the manufacturer. Purified DNA (10 ng) was used as template for all PCR reactions. The PCR conditions and primers used in this study are described in Table 2.

**Table 2:** PCR conditions, nucleotide sequences and *amplicon* sizes for the specific *Campylobacter jejuni primers* used in this study.

**Genes**	**Primers**	**Sequence 5′ → 3′**	**Size (pb)**	**DNA (ng)**	**Primer (pmol)**	**PCR Conditions**	**References**
*flaA*	flaA-F	ATGGGATTTCGTATTAACAC	1728	50	10	94°C – 10 min; 30 cicles: 94°C – 1 min, 47°C – 1 min, 72°C – 1 min; 72°C – 10 min	Hanel et al., 2004
	flaA-R	CTGTAGTAATCTTAAAACATTTTG					
*cadF*	cadFI-F2B	TTGAAGGTAATTTAGATATG	400	40	40	94°C – 10 min; 30 cicles: 94°C – 1 min, 47°C – 1 min, 72°C – 1 min; 72°C – 10 min	Zheng et al., 2006
	cadFI-R1B	CTAATACCTAAAGTTGAAAC					
*luxS*	luxS-1	AGGCAAAGCTCCTGGTAAGGCCAA	1080	50	10	94°C – 3 min; 30 cicles: 94°C – 30 s, 55°C – 1 min, 72°C – 1 min; 72°C – 10 min	Elvers and Park, 2002
	luxS-2	GGATCCGTATAGGTAAGTTCATTTTTGCTCC					
*dnaJ*	dnaJ F	AAGGCTTTGGCTCATC	720	20	20	95°C – 2 min; 30 cicles: 94°C – 1 min, 46°C – 1 min, 72°C – 1 min; 72°C – 5 min	Datta et al., 2003
	dnaJ R	CTTTTTGTTCATCGTT					
*htrA*	htrA F	TAATACGACTCACTATAGGGTAAGTTTAGCAAGTGCTTTATTTGC	1393	10	10	95°C – 1 min; 35 cicles: 95°C – 30 s, 50°C – 1 min, 72°C – 1 min; 72°C – 5 min	Datta et al., 2003
	htrA R	AAAACCATTGCGATATACCCAAACT					
*cbrA*	cbrA F	TAATACGACTCACTATAGGGTCAACTCTATCCTTGCCATTATCTT	1165	10	10	95°C – 1 min; 35 cicles: 95°C – 30 s, 50°C – 1 min, 72°C – 1 min; 72°C – 5 min	Biswas et al., 2011
	cbrB R	GTAGATATTGCTTTTGGTTTTGCTG					
*sodB*	sodB F	ATGATACCAATGCTTTTGGTGATTT	638	20	20	95°C – 2 min; 30 cicles: 94°C – 1 min, 46°C – 1 min, 72°C – 1 min; 72°C – 5 min	Biswas et al., 2011
	sodB R	TAATACGACTCACTATAGGGCATTTGCATAAAAGCTAACTGATCC					

The studied genes were *flaA* (motility), *cadF* (intracellular colonization), *luxS* (quorum-sensing mechanism), *dnaJ* (thermotolerance), *htrA* (aids in growth under stress), *cbrA* (resistance to osmotic shock), and *sodB* (tolerance to oxidative stress).

PCR reactions were performed using the GoTaq® Green Master Mix kit (Promega) according to the manufacturer's instructions. The amplified products were subjected to 1.5% agarose gel electrophoresis using the TBE 0.5x runner buffer (Invitrogen) and as a molecular weight standard of 100 pb marker (Invitrogen).

### Biofilm inhibition test

To examine the interaction between *C. jejuni* biofilms with biocides components and nanoparticles, the protocol described by Lu et al. (2012) was used. The chemical compounds tested were: Chlorhexidine 1% solution, Sodium Hypochlorite 1%, Peracetic Acid 0.8% and Zinc Oxide (ZnO) nanoparticles 6 mmol/L.

Ten colonies grown in CCDA plates were diluted in 10 mL of 0.9% NaCl solution, adjusted according Mc Farland scale 0.5. From this solution, a 100 μL aliquot (corresponding to 10^7^ cells), was inoculated onto a sterile cellulose membrane with 0.45 μm of porosity and 47 mm of diameter on a *Brucella* agar plate (Difco) enriched with 5% of defibrinated sheep blood. The plates were incubated at 37°C in microaerophilia and in every 24 h the membrane was transferred to a new plate, during 3 days.

Subsequently, the membrane was placed in a flask containing 20 mL of MH broth with respective concentrations of the chemical compounds. The flasks were incubated in microaerophylia at 37°C for 24 h. Subsequently, the membrane was washed three times with phosphate buffer (PBS), followed by treatment in 25 mL of 0.1% trypsin for 15 min at room temperature. Thereafter, the resulting solution of the incubation underwent serial dilutions for further counting.

### Biofilm stability test

The biofilm stability assay was performed according to the protocol described by Chaignon et al. (2007), with some modifications. Biofilms were formed into 96-well plates as described above. After 48 h of growth, the culture medium was removed, the wells were washed twice with sterile 0.9% NaCl solution and then filled with 200 μl of a proteinase K solution (Invitrogen, USA) on concentration of 1 mg/ml in 20 mM Tris (pH 7.5) and 100 mM NaCl or 200 μl of a 10 mM sodium metaperiodate solution (Sigma-Aldrich, USA) prepared in 50 mM acetate buffer (pH 4.5). The plates were incubated for 2 h at 37°C. After treatment, the biofilms were washed with 200 μL of sterile 0.9% NaCl and stained with 1% crystal violet. The absorbance was evaluated on a plate reader at 595 nm with an alcohol-ketone solution containing 80% of ethanol and 20% of acetone (Synth®), as white. The experiment was performed in biofilms of three *C. jejuni* strains formed with chicken juice. All assays were performed in eight wells, on three independent occasions.

### Scanning electron microscopy

The preparation of the material for analysis in SEM was done according to Brown et al. (2014) with modifications. Simple and mixed biofilms in the MH and chicken juice media were formed in glass beads with a diameter of 5 mm, respecting the growth conditions described for *Campylobacter*: microaerophilic atmosphere at 37°C for 48 h. After biomass formation, the samples were fixed with 2.5% glutaraldehyde and 2.5% paraformaldehyde in 0.1 M buffer PBS (pH 7.4) overnight at 4°C. The fixative was removed, and the samples washed three times with PBS buffer. The beads were post-fixed with 1% osmium tetroxide for 2 h and washed three times with PBS buffer. The beads were dehydrated in a series of ethanol solutions (30, 40, 50, 60, 70, 80, and 90% and then three times at 100%) for 20 min for each step.

The samples were dried in CPD (Critical Drying Point) (CPD 030, Baltec, Germany) using liquid carbon dioxide as the transition fluid. The samples were coated with a 20 nm thick layer of gold (SCD 050, Baltec, Germany) and visualized on MEV VP Zeiss Supra 55 SEM FEG operating at 5 kV.

### PFGE

The isolates were typed by PFGE according to the protocol described in the Center Disease and Control (2013). Digestion of the intact genomic DNA was done with 30 U of the enzyme *Sma* I (Invitrogen) for 2 h at 25°C. The DNA fragments were separated on 1% agarose gel (SeaKem Gold) in 0.5X TBE buffer in the CHEF DRIII (Bio-Rad) apparatus, for a period of 18 h, with the following parameters, 200 v, 120° angle, Gradient of 6 v/cm and buffer temperature of 14°C.

The gels were stained with ethidium bromide and photographed under UV light. The analysis for dendrogram formation was performed using GelCompare II software. The comparison of the band patterns was performed by the UPGMA analysis method, using the Dice similarity coefficient.

### Statistical analysis

The obtained results were analyzed using GraphPad Prism, version 6.0. Qualitative and quantitative biofilm formation tests were evaluated using simple variance analysis (ANOVA). For the biofilm inhibition test, ANOVA was used to compare the results of the control with the resistant strains in the test groups, and to analyze the counts between the control strains and tests separately. For the simple comparisons of two variables, in the biofilm stability test, Student's *t*-test was used. All tests were performed at a confidence level of 95%.

## Results

### Ability to adhere to the abiotic surface

The adhesion assays were conducted in Mueller Hinton and Muller Hinton supplemented with chicken juice media with an initial bacterial concentration of approximately 10^3^ CFU/well. The indices found showed that the ability to adhere to the polystyrene varied according to the strain.

The results showed that all tested strains had adhesion capacity when inoculated in Muller Hinton (MH) and MH + 5% of chicken juice, but there was a reduction in counts (*p* < 0.05) compared to the initial inoculum when the strains were held at Mueller Hinton.

**Figure 1:**
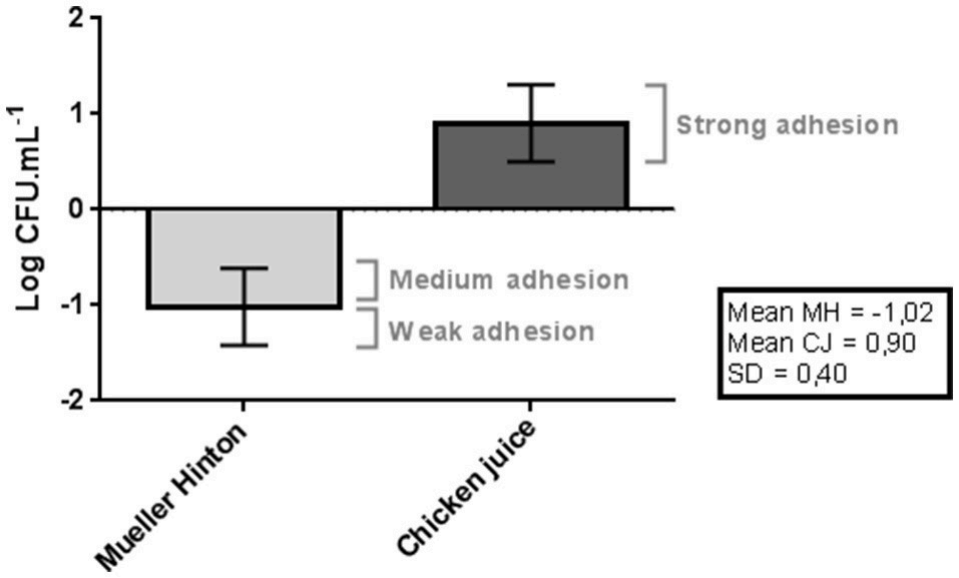
Difference between adhesion and the mean of initial inoculum obtained in the counts (log of CFU/mL) of *C. jejuni* in the Mueller Hinton and Chicken juice. Error bars indicate the standard deviation for the means of the counts obtained for each strain at three repetitions. SD indicates the standard deviation used in the classification of the strains: Poor adherence (counts below the mean in MH up to 1 SD), Average adherence (higher than average counts in MH up to 1 SD), Strong adherence (higher than average counts in MH Up to more than 1 SD).

In Mueller Hinton, 46.7% (14/30) of the strains showed medium adhesion pattern, with a count above the average in all the tests, and 53.3% (16/30) of the strains showed weak adhesion by presenting lower values than those obtained in the general mean of the initial inoculum (*p* < 0.05), as shown in Figure 1.

The values were significantly higher (*p* < 0.05) when the strains were inoculated in the chicken juice, with all 30 (100%) strains classified as strongly adherent, because they had higher counts than the initial inoculum.

The ability of these strains to adhere strongly in conditions similar to those present in the avian industry (chicken juice) helps to explain their survival and persistence in the slaughterhouse. The 4-h incubation period was sufficient for the initial establishment of the biofilm structure, and thus could act as a constant source of contamination in the industry.

The nutritive particles available in chicken juice can form a thin layer above the surface of the polystyrene wells and on glass surfaces that facilitate this bacterial adhesion (Li, 2016).

### Classification and quantification of *C. jejuni* biofilms

All of *C. jejuni* strains (100%) were capable of forming strong biofilms when supplemented with chicken juice by the crystal violet test (Table 3). The same did not occur when in the presence of Mueller Hinton, where none of the strains presented a strong producer profile under this condition. The inclusion of chicken juice promoted a significant increase in bacterial biomass, increasing in average 1.70 the Biofilm Formation Index (BFI) when compared to the value found in non-supplemented samples.

**Table 3:** Classification of strains, according to the BFI (Biofilm Formation Index), under the different enrichment conditions.

**Identification**	**Mueller hinton (MH)**		**MH + 5% of Chicken juice**	
	**BFI**	**Classification**	**BFI**	**Classification**
F 03	0.476	Weak	1.926	Strong
F 45	0.261	Nonexistent	2.199	Strong
F 48	0.119	Nonexistent	2.243	Strong
F 51	0.280	Nonexistent	2.105	Strong
F 67	0.721	Medium	2.2665	Strong
F 68	0.388	Weak	2.558	Strong
F 80	0.326	Nonexistent	2.114	Strong
F 84	0.625	Weak	2.226	Strong
F 85	0.417	Weak	2.086	Strong
F 87	0.310	Nonexistent	1.937	Strong
F 88	0.482	Weak	1.988	Strong
F 100	0.304	Nonexistent	1.632	Strong
F 127	0.897	Medium	2.338	Strong
F 138	0.576	Weak	2.362	Strong
F 140	0.847	Medium	2.190	Strong
F 157	0.841	Medium	2.302	Strong
F 163	0.393	Weak	2.377	Strong
F 164	0.560	Weak	2.343	Strong
F 172	0.513	Weak	2.344	Strong
F 175	0.491	Weak	2.165	Strong
F 206	0.320	Nonexistent	2.178	Strong
F 211	0.327	Nonexistent	2.437	Strong
F 236	0.677	Weak	1.864	Strong
F 240	0.917	Medium	2.658	Strong
F 246	0.492	Weak	2.141	Strong
F 247	0.970	Medium	2.339	Strong
F 248	0.266	Nonexistent	2.143	Strong
F 253	0.325	Nonexistent	2.429	Strong
F 255	0.644	Weak	2.423	Strong
F 256	0.964	Medium	2.761	Strong

In the condition not supplemented with chicken juice, 10/30 (33.3%) strains did not form biofilms, 13/30 (43.3%) were classified as weak producer and 7/30 (23.3%) as medium producer, according to Table 1.

These data shows that surfaces in contact with organic matter inside the industry may harbor these biofilms, since there is a constant presence of chicken juice during the processing of chicken carcasses. If hygiene measures are not frequent and sufficiently conducted, the exudate present in the chicken carcass guarantees conditions for *C. jejuni* maintenance.

The data obtained in the biofilm counts proved the differences obtained in the tests with crystal violet. Starting from a constant initial inoculum (*p* > 0.05) in all assays, it was observed that in both, the adhesion and biofilm formation, had a significant increase in the counts and in bacterial multiplication in the presence of chicken juice when compared to the counts in Mueller Hinton (Table 4).

**Table 4:** Counts obtained in the assays for adhesion analysis and biofilm formation in the 30 strains of *C. jejuni*.

**Mediums**	**Mean counts ± standard deviation (Log CFU/mL)**		
	**Initial inoculum**	**Adhesion**	**Biofilm**
Mueller Hinton	4.15 ± 0.29 Aa	3.13 ± 0.35 aB	5.30 ± 0.38 aC
Chicken juice	4.10 ± 0.32 Aa	5,00 ± 0.49 bB	7.37 ± 0.23 bC

*Different lowercase letters in the columns and different capital letters on the lines indicate significant difference*.

The results obtained in the adhesion assays showed that in relation to the initial inoculum there was a significant reduction in the counts when the strains were inoculated in MH and a significant increase in the chicken juice (*p* < 0.05). This fact reveals the difficulty of maintaining and developing the initial structure of the biofilm in Mueller Hinton.

The values obtained in the adhesion (4 h) in chicken juice were similar to those found after 48 h (biofilm) in MH, indicating another evidence of the superiority of chicken juice in the establishment of the sessile form of *C. jejuni*. In addition, the high counts detected in chicken juice after biofilm formation, suggests that in this condition the biofilm may be well established.

### Genetic repertoire related to sessile form of *C. jejuni*

Analysis of the seven genes potentially required for the formation of strong biofilms in *C. jejuni* showed that all strains (100%) have the potential to form strong biofilms, since all the genes evaluated were identified in the 30 strains.

These findings are consistent with the results obtained in the counts and the colorimetric test (crystal violet) of the biofilms of *C. jejuni* in the presence of chicken juice. This supplement probably allowed expression of genes associated with the transition to sessile form, including the studied genes.

### Chemical agents reduce *C. jejuni* biofilm

The use of chemical agents had a high potential for elimination of viable cells from *C. jejuni* biofilms. For all the used products, was a significant reduction in the bacterial counts in relation to the untreated biofilm. In 17/30 (56.7%) of biofilms, total elimination of the microorganism was observed for all chemical agents tested.

**Figure 2:**
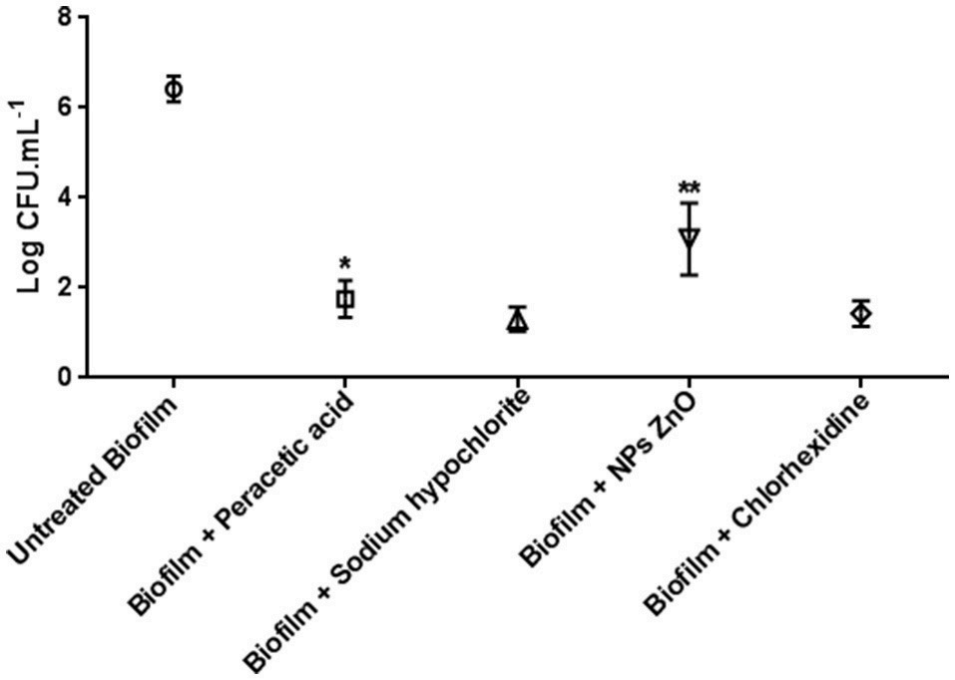
Biofilm counts of *C. jejuni* (log CFU/mL) in the control test (30 strains) and maintained for 24 h in peracetic acid solution 0.8% (7 strains), sodium hypochlorite 1%, ZnO NP 6 mM (13 strains), and chlorhexidine 1% (4 strains). ^*^p < 0.01; ^**^p < 0.001 using one way Anova for the counts in the samples of the same treatment.

Figure 2 shows the counts obtained in the untreated biofilms, which obtained a mean value of 6.41 Log CFU/mL (*p* > 0.05), and the resistant biofilms treated with the different products that showed growth after 24 h in contact.

The inclusion of sodium hypochlorite 1% allowed the survival of 6/30 (20.0%) of the strains, with a mean count of 1.30 log CFU/mL. This value did not differ between strains (*p* > 0.05), and demonstrated a reduction of about 5.11 log cycles relative to untreated biofilm.

There was also no difference in counts after the use of chlorhexidine 1% (*p* > 0.05) for 4/30 (13.3%) strains tolerant to this agent. The mean count of 1.33 log CFU/mL after treatment showed a mean decrease of 5.08 log cycles compared to the control.

For peracetic acid and ZnO nanoparticles (NPs), the reduction in the number of CFUs varied significantly (*p* < 0.05) among the tolerant strains, indicating that persistence in the presence of these agents may be a characteristic strain-dependent.

Seven of the 30 strains (23.3%) in the sessile form survived in the presence of peracetic acid with counts varying from 1.34 to 2.16 log of CFU/mL. Therefore, the reduction was from 4.25 to 5.07 log cycles compared to the control.

In the presence of ZnO NPs, it was observed that 13/30 (43.3%) strains were tolerant and presented the highest log of CFU/mL, alternating from 2.09 to 4.07. The decrease was from 2.34 to 4.32 log cycles in relation to the control.

In general, chlorhexidine 1%, sodium hypochlorite 1% and peracetic acid 0.8% presented equivalent efficiency in the control of *C. jejuni* biofilm (*p* > 0.05), due to the high number of biofilms removed and by the low counts obtained for the resistant strains. ZnO NPs presented the lowest treatment efficacy (*p* < 0.05).

Table 5 shows the resistance profiles of the agents obtained for the 13 strains that showed growth after the biofilm inhibition test.

**Table 5:** Resistance profiles to chemical agents tested on 13 biofilms of *C. jejuni*.

**Chemical agents**				**Profile**	**Number of strains**
ZnO NPs				I	4 (30.8%)
ZnO NPs	Chlorhexidine			II	1 (7.7%)
Peracetic acid	ZnO NPs			III	2 (15.4%)
Hypochlorite	ZnO NPs	Chlorhexidine		IV	1 (7.7%)
Peracetic acid	Hypochlorite	ZnO NPs		V	3 (23.1%)
Peracetic acid	Hypochlorite	ZnO NPs	Chlorhexidine	VI	2 (15.4%)

The presence of biofilms resistant to disinfectant agents shows that there are probably intrinsic or extrinsic adaptive mechanisms that allow their survival. According to Table 5, two strains showed resistance profile VI (F206 and F246), that is, they are tolerant to all agents and may characterize a problem in the industry due to the difficulty of eliminating them and the risk of dissemination of this characteristic to other strains.

### Structure and composition of *C. jejuni* and mixed biofilms

**Figure 3:**
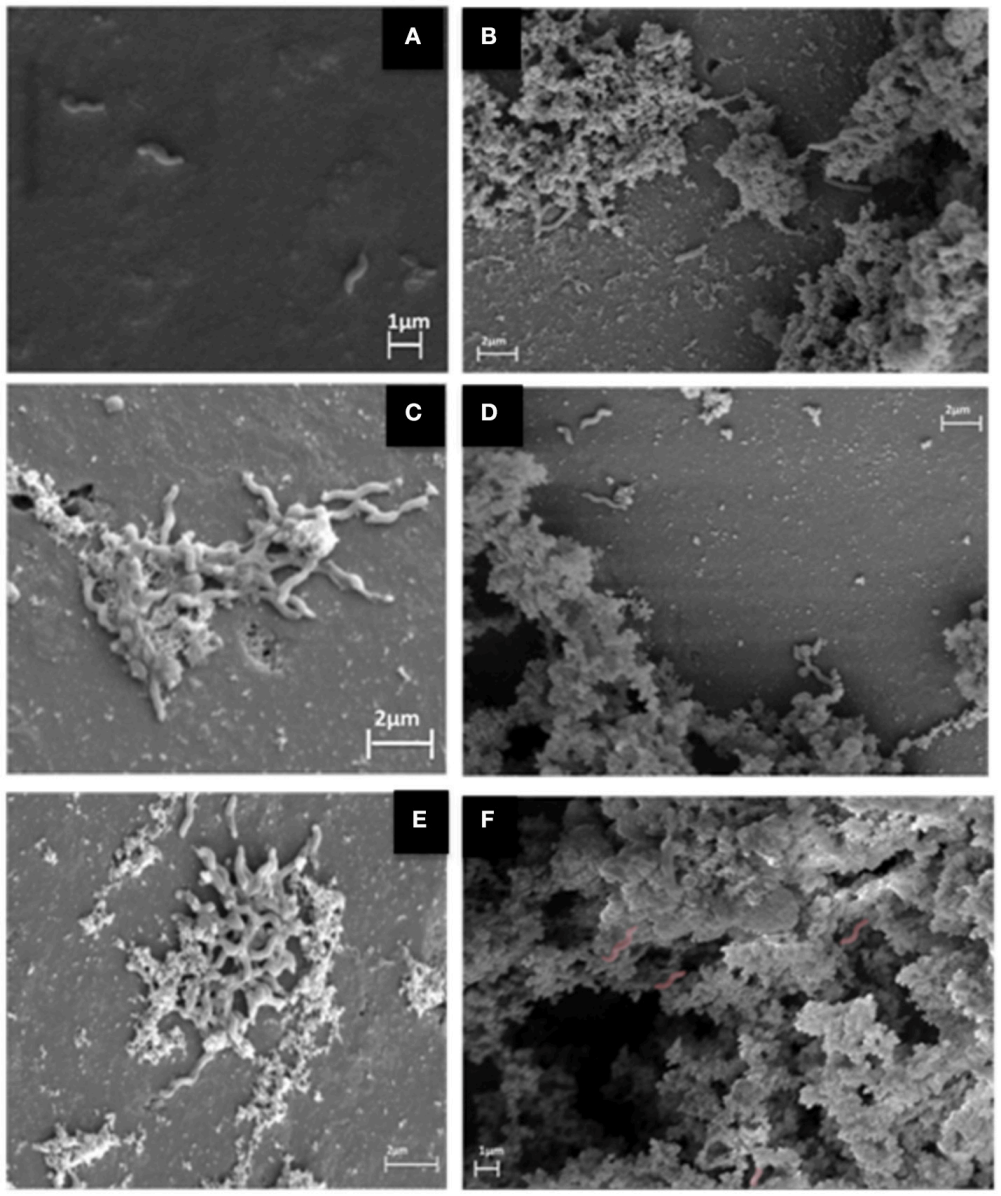
SEM images for three *C. jejuni* strains with different BFI (biofilm formation index) in MH (**A**, nonexistent; **C**, weak; and **E**, medium), and strong producers in chicken juice **(B,D,F)** (Computerized staining in *C. jejuni* in **F**).

At the SEM assay we observed changes in biomass formed for the three types of biofilm patterns identified in the MH: F80 (unable to produce biofilm), F255 (weak producer) and F256 (medium producer). In addition, difference in yield was noted when the substrate for its growth was supplemented with chicken juice (Figure 3).

Figure 3A shows the presence of isolated bacteria, indicating the inability to form biofilm in this condition. In 4c and 4e the initial formation of biofilm is observed, with primary production of extracellular matrix. Already in 3b, 3d, and 3f (in chicken juice) there is formation of the mature biofilm, with a tridimensional structure of the evident matrix.

**Figure 4:**
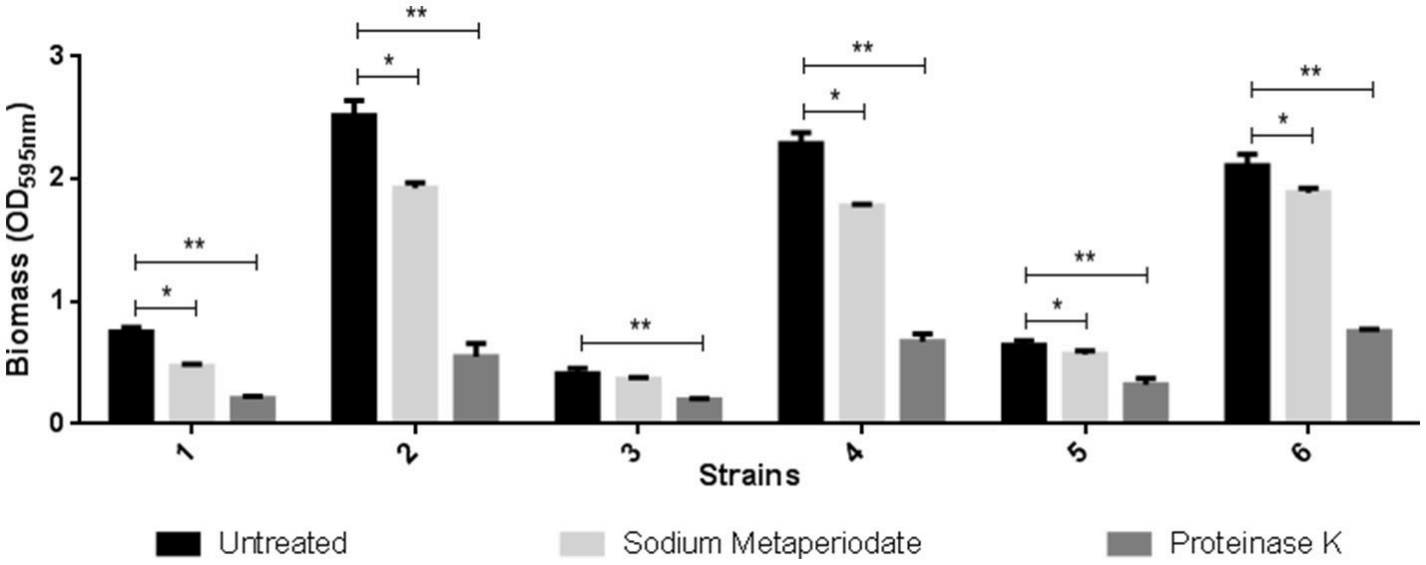
Effect of treatment with sodium metaperiodate and proteinase K on the biofilm of three *C. jejuni* strains. Results represent means with standard deviation (error bars) of three independent experiments. 1 (F 80 in Mueller Hinton), 2 (F 80 in chicken juice), 3 (F 255 in Mueller Hinton), 4 (F 255 in chicken juice), 5 (F 256 in Mueller Hinton), and 6 (F 256 in Chicken juice). ^*^p < 0.05; ^**^p < 0.001 using one way Anova.

The composition assay performed with proteinase K and sodium metaperiodate promoted protein degradation and carbohydrate oxidation (Figure 4).

In both MH and chicken juice biofilms, the proteinase treatment almost completely removed the biomass formed by the three strains tested. However, the carbohydrate oxidant showed little or no effect (F255 in MH) on the biofilm produced by these strains.

**Figure 5:**
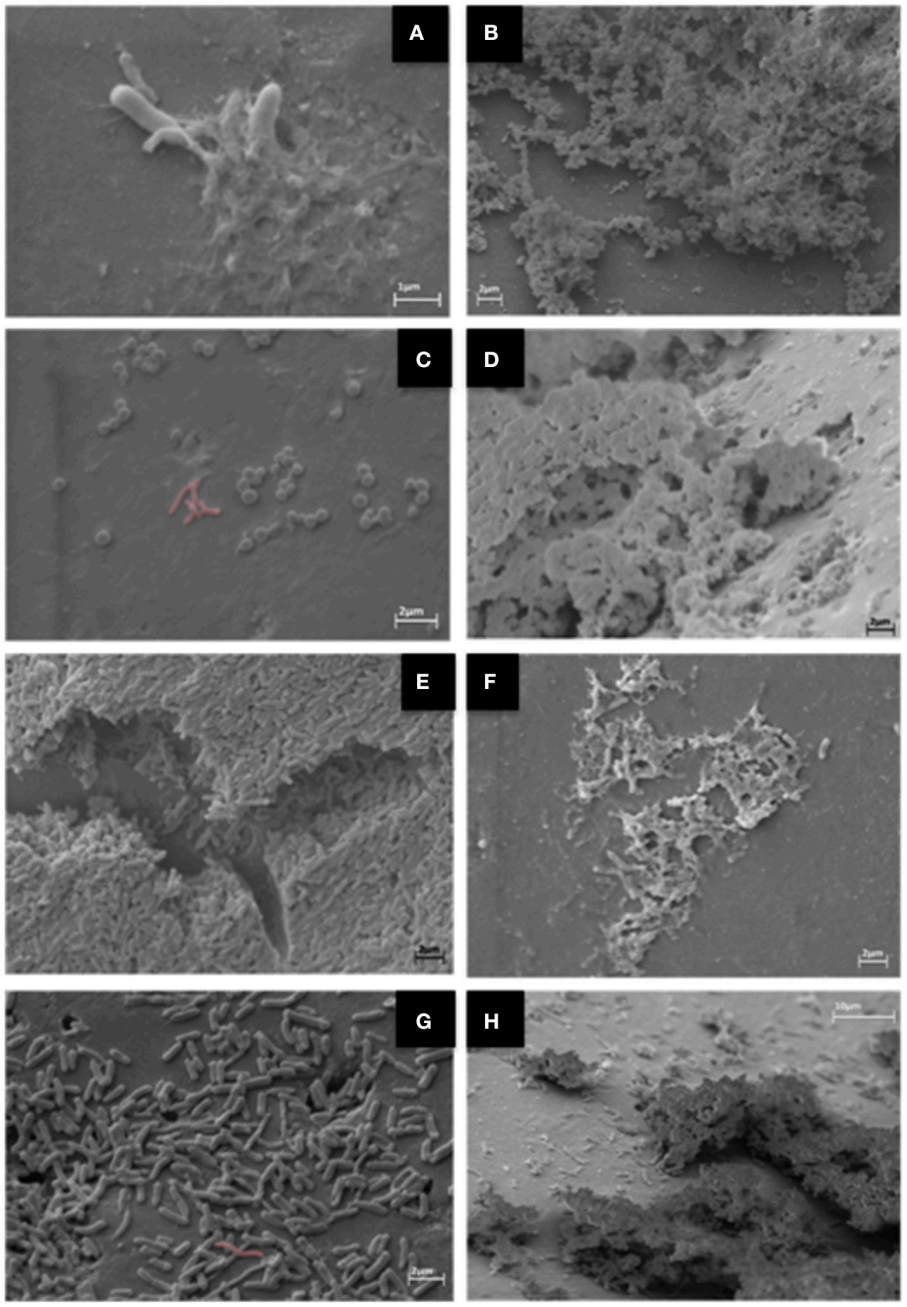
SEM images for mixed biofilms. *C. jejuni* with *Salmonella* Enteritidis in MH **(A)** and in Chicken Juice (CJ) **(B)**, with *Staplylococcus aureus* in MH **(C)**, and in CJ **(D)**, with *Pseudomonas aeruginosa* in MH **(E)** and in CJ **(F)** and with *Escherichia coli* in MH **(G)** and in CJ **(H)**. Computerized staining in *C. jejuni* in **(C)** and **(G)**.

For the mixed biofilm assays of *C. jejuni* with *Escherichia coli, P. aeruginosa, Salmonella* Enteritidis and *Staphylococcus aureus* the SEM demonstrated competitive disadvantage of the three strains of *C. jejuni* tested. The predominance of the other species was clear as shown in Figure 5, indicating the selection pressure exerted by the other species on *C. jejuni*.

**Figure 6:**
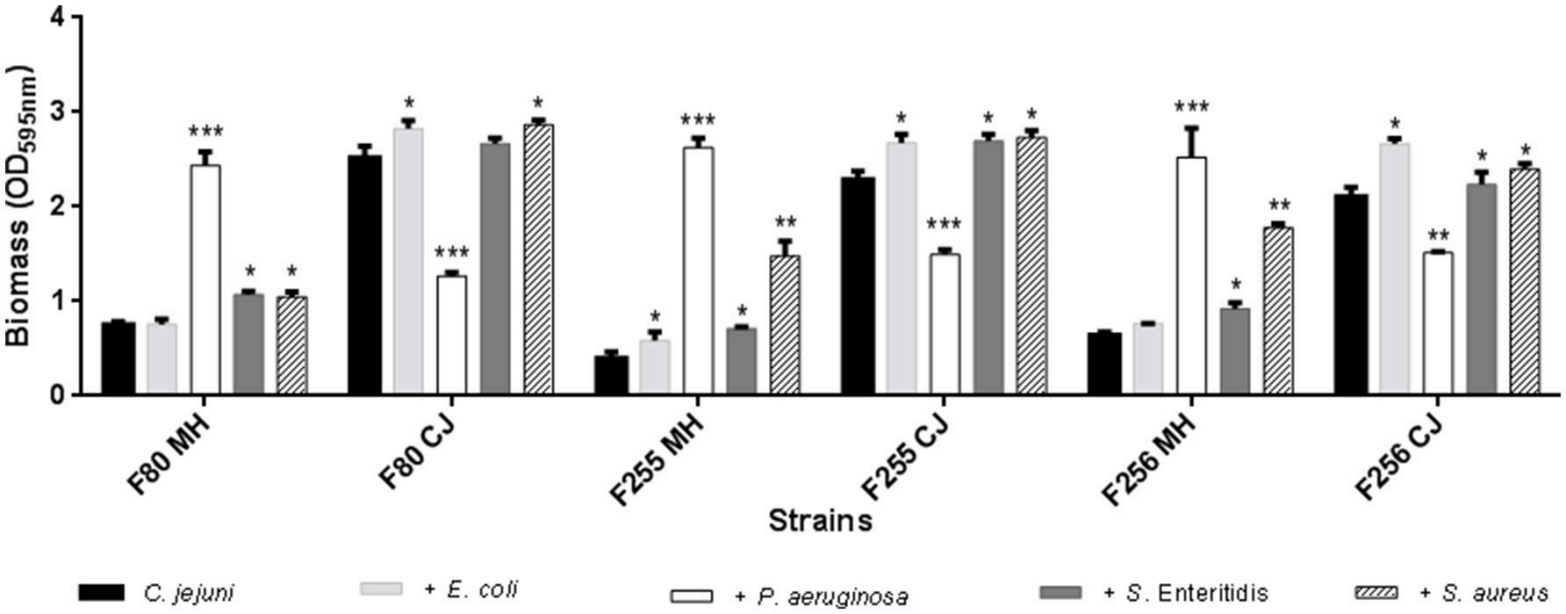
Changes in biomass of mixed biofilms of *C. jejuni* with *E. coli*, with *P. aeruginosa*, with *S. Enteritidis* and with *S. aureus*, separately. Results represent means with standard deviation (error bars) of three independent experiments. ^*^p < 0.05; ^**^p < 0.01; ^***^p < 0.001 using Student's *T*-test for comparisons with the control (*C. jejuni*).

In Figure 6 was possible to verify the alteration in the biomass formed by the crystal violet method compared to the control group composed of simple biofilm of only *C. jejuni*.

In practically all the tests, there was a higher production of biomass in relation to the control. The exception is in the mixed biofilms with *P. aeruginosa* that exhibited a different behavior from that found for the other species. In the presence of MH, the biofilm production was exacerbated (*p* < 0.001), but in chicken juice the biomass was significantly lower, showing that some factor present in chicken juice could inhibit the transition to sessile form in this specie.

This fact was also observed in SEM, by the formation of a denser biomass in MH when compared to chicken juice (Figures 5E,F).

The assays concerning to the composition of the matrix for mixed cultures are probably more related to the other species and not, in fact, to *C. jejuni*. For all mixed biofilms the composition was predominantly proteic, except for mixed biofilms with *P. aeruginosa* whose presence of carbohydrates was more evident (data not shown).

### Genetic diversity of *C. jejuni*

**Figure 7:**
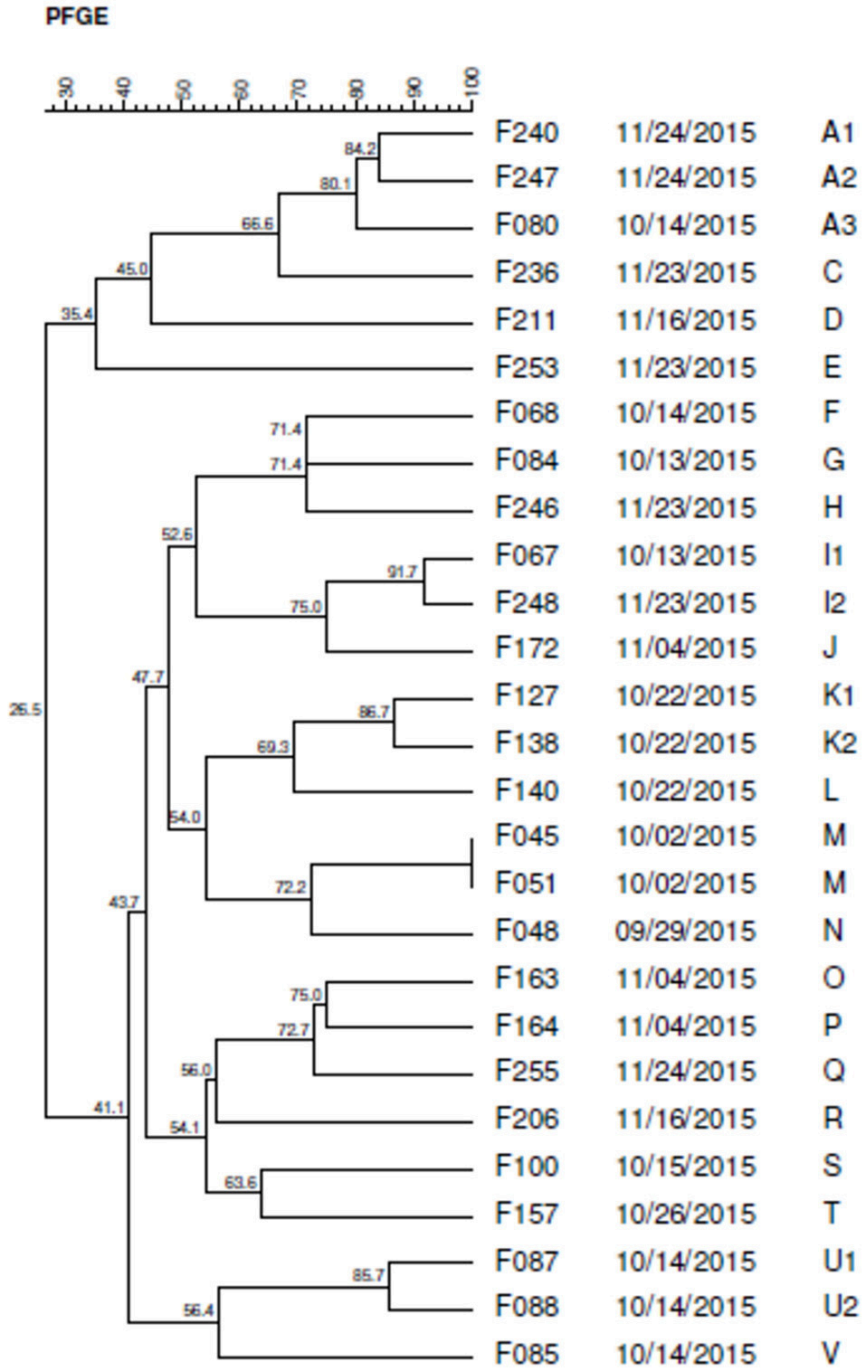
Dendrogram generated by computerized analysis (Gel Compare II) of DNA profiles of *C. jejuni* strains, based on pulsed field electrophoresis (PFGE). The analysis was performed by the Dice/UPGMA method (tolerance parameter of 0.5%, optimization of 0.5%, homology ≥ 80%).

Twenty three pulsotypes (A-V) were identified by PFGE, being 17 of them characterized as distinct profiles (Figure 7).

Five profiles (A, I, K, M, and U) were classified as clusters with homology higher than 80%, composed of strains with the same genotype. The M-type pulse was designated as a clone because it showed 100% of similarity.

The K, M, and U pulsotypes, grouped isolated strains on the same date, indicating possible cross-contamination among the samples. However, the A and I pulses presented isolated strains at different dates suggesting the persistence of this genotype in the industry, probably due to the biofilm formation.

## Discussion

### Biofilms of *C. jejuni*

During the last decade, *C. jejuni* has been regularly presented as the leading cause of bacterial foodborne infections in Europe and the USA. Given the importance to public health of this zoonosis, it is relevant to understand the survival mechanisms adopted by this pathogen.

One of the mysteries of the genus *Campylobacter* is that it is a pathogenic microorganism that survives successfully in the host and industrial environment under stressful conditions, and paradoxically is a mandatory microaerophilic that survives poorly under controlled laboratory conditions. In addition, in comparison to other agents causing foodborne disease, such as *E. coli* and *Salmonella* spp., *C. jejuni* needs a low infective dose (500–800 CFU) to cause disease in the host (Black et al., 1988). Although this may contribute to infection, it is still unclear what allows the bacteria to survive during transmission under adverse conditions.

Survival in a biofilm would be an explanation to protect bacteria from various environmental stresses, antimicrobial agents and/or disinfectants and the immune response of the host.

In this study we found that these structures represent a reservoir of cells and that the level of biofilm formation by *C. jejuni* is clearly increased under conditions similar to those found in the industry with the presence of chicken juice.

The detection of viable cells in significant quantities in biofilms formed in chicken juice corroborates the idea that survival and persistence in the production chain may represent the main problem of contamination in final product. Despite the use of microaerophylia for this study, it is known that the mature biofilm can provide an adequate environment for microaerophilic growth allowing the ideal conditions for maintenance and dissemination of this pathogen (Reuter et al., 2010).

The biofilm formation involves the succession of several steps, starting with initial adhesion. For this reason, *C. jejuni*'s ability to adhere to a inert surface was investigated, in order to subsequently assess their ability to initiate and develop the biofilm. The adhesion capacity was variable and lower in the 30 strains tested in MH. The delayed adhesion profile may indicate less ability to acquire the sessile form, but may also be related to the need for a prolonged period of contact with the surface to lead to a stronger future adhesion (Turonova et al., 2015). In contrast, in chicken juice the counts showed high adhesion capacity for all strains. The medium supplemented with chicken juice allowed a better condition for adhesion to the inert surface (Li, 2016).

The results obtained in both colorimetric and quantitative tests revealed the superiority of chicken juice in relation to MH.

Chicken carcass exudates contain a complex mixture of carbohydrates, proteins, lipids, and sugars (Chmielewski and Frank, 2007), providing an ideal medium for the proliferation and survival of bacteria. The accumulation of these organic materials allows the formation of micro-layers on the surfaces that aid in bacterial fixation, together with greater availability of nutrients (Hwang et al., 2012).

Thus, in the industrial environment, the presence of meat-based exudates may exacerbate the problem of contamination by *C. jejuni*. Our results add and are consistent with the findings of Brown et al. (2015) who also detected the efficiency of chicken juice at different concentrations in the biofilm production for five *Campylobacter* strains.

### Genetic apparatus of *C. jejuni*

Once the phenotypic characterization was performed concerning the sessile kind of living, analysis of the specific genes revealed that all strains possess the genes required to develop a biofilm.

Thus, gene identification in the strains of *C. jejuni* did not explain the differences in the classification of the biofilms formed in MH. In contrast, the identification of all the genes surveyed in all strains is consistent with the strong producer character obtained in chicken juice. Therefore, chicken juice is likely to provide all the necessary conditions for expression of the genetic potential recorded by the presence of *flaA, cadF, luxS, dnaJ, htrA, cbrA*, and *sodB* genes and this same ability is not detected in MH.

The genes linked to quorum-sensing, adhesion, adverse conditions and motility were all previously described as important for the acquisition of the sessile form (Kalmokoff et al., 2006; Svensson et al., 2009; Howlett et al., 2012; Sulaeman et al., 2012; Avila-Ramirez et al., 2013; van Alphen et al., 2014).

There are reports that flagellar expression is required for the formation of biofilms by *C. jejuni* (Lehtola et al., 2006; Reeser et al., 2007), including *flaA* and *flaB* genes (Reuter et al., 2010). However, the absence of these characteristics does not completely prevent the acquisition of the sessile form. The advantage in the expression of this characteristic is due to the initial fixation, biofilm structuring, orientation to a pre-existing biofilm in addition to the correlation with other non-flagellar extracellular proteins that contribute indirectly to the sessile lifestyle (Howard et al., 2009; Kim et al., 2015).

Numerous genes in *Campylobacter* were previously described as mediators of adhesion *in vitro*. Among them, the *cadF* gene that encodes the binding proteins CadF fibronectin (Konkel et al., 2010).

Several enzymes and proteins are already described by the involvement in bacterial protection against oxidative stress, whose action is related to peroxide or superoxide detoxification. Among them, the enzyme superoxide dismutase (SodB) appears as a major regulator in *C. jejuni* (Flint et al., 2014; Kim et al., 2015).

Some quorum-sensing systems have already been detected in *Campylobacter*. The production of AI-1 (acyl-homoserine autoinducer) represents one of these mechanisms, which accumulates in the extracellular environment and diffuses freely in the bacterial cytoplasm, which at high levels binds to a cellular transcription enhancer (*luxS*) that encodes the luciferase, a metabolic key enzyme in the SAM recycling pathway (S-adenosylmethionine). This metabolite is essential in the performance of important biosynthetic reactions, such as the methylation of bacterial DNA, the synthesis of polyamines and bacterial vitamins. The most important performance of the *luxS* gene is associated with the synthesis of a new AI called autoinducer-2 (AI-2). Increased bacterial population growth also promotes elevation of AI-2 concentrations in the environment. The *luxS* gene acts in the formation of several molecular compounds, which together are called AI-2 variants. These molecules have potential for recognition and inclusion of mixed populations and of the same species in biofilms (Xavier and Bassler, 2005).

Much of *C. jejuni* has functional LuxS enzymes and is capable of producing AI-2. However, the presence of nutrients is necessary for the production of AI-2, and these are found in foods, such as milk and chicken juice, even when the microorganisms are kept under adverse conditions, such as in oxidative stress and in low temperatures (Ligowska et al., 2011; Parveen and Cornell, 2011; Tazumi et al., 2011; Plummer, 2012).

### Strategies for the elimination of viable cells of sessile *C. jejuni*

In the poultry industry investigated, the chemical agents: peracetic acid 0.8%, sodium hypochlorite 1% and chlorhexidine 1%; are used by the quality control team. On the other hand, ZnO NPs, represent a potential sanitizing agent for experimental use, with no usual application in hygiene in the food producing industries.

The results showed that the three agents used in the industry routine were more effective in elimination, although 9/30 (30.0%) of the strains were identified to be tolerant to at least one of them. In contrast, ZnO NPs showed less efficacy with 13/30 (43.3%) resistant strains and with counts higher than the other agents.

The presence of tolerant strains to different sanitizers suggests that the use of these agents in the routine of the industrial environment in an inadequate way can result in the sublethal exposure to these biocides, representing a real risk for the adaptation of these bacteria, besides positively influencing the production of biofilms (Keeratipibul and Techaruwichit, 2012; Techaruvichit et al., 2016).

As for ZnO NPs it is possible that tolerant bacteria have already acquired characteristics that confer this resistance, such as the presence of efflux pumps, ZnO resistance genes and the ability to maintain intact the integrity of membrane. This characteristic has already been identified in *Escherichia coli* and *Enterococcus faecium* (Mileyeva-Biebesheimer, 2011).

Although the use of chemical compounds provides benefits in disinfection, they have the limitation of not destroying the residual structures of the biofilm matrix that may facilitate their resurgence or maintenance (Ohsumi et al., 2015). Thus, special efforts are required for the complete removal of highly adherent biofilms adapted to *C. jejuni* biocides (Techaruvichit et al., 2016).

Probably, the effectiveness in the control is possible by the association of hygiene plans with different agents, respecting the periods between cleaning, besides strategies, like the periodical rotation of biocides.

### Architecture and constitution of *C. jejuni* and mixed biofilms

For the three *C. jejuni* strains under sessile form in the glass beads, with MH substrate plus chicken juice, it was observed in SEM that the structure of the biofilm was quite similar, with a more expanded and stable architecture, besides the presence of irregular coverage along the surface of the sphere, consistent with the presence of several macrocolonies. Differently, in MH, this pattern varied according to the strain, so that the most developed structure observed was the presence of microcolonies that indicate the immature stage of the biofilm.

A study by Bronnec et al. (2016) compared the ultrastructure of two strains of *C. jejuni* in biofilm under microaerophilic and aerobiose conditions. The authors concluded that the differences revealed the formation of mature and immature biofilm, being a strain-dependent characteristic.

The variations in the architecture of the formed biofilms can have relation not only with the nutrient available to the bacterium, but also because it is a strain-dependent character. Turonova et al. (2015) showed that *C. jejuni* NCTC 11168 produces biofilm with multilayer type structure, while *C. jejuni* 81–176 was able to form finger-like biofilm with an open ultrastructure.

The capacity to form biofilm with open ultrastructure composed of wells and channels was identified in the three strains of *C. jejuni* tested in the presence of chicken juice. This type of heterogeneous structure gives the characteristic of a mature biofilm, which allows the formation of interconnected fluxes that aid in the access to nutrients for the cellular aggregates and in the drainage of the metabolic residues (Donlan and Costerton, 2002).

The composition assays allowed to identify that all strains reduced biomass with treatment with sodium metaperiodate and proteinase K, the last one being more significant. Thus, the treatment of biomass with products of proteolytic action can be considered an effective mechanism for partial degradation, allowing a better penetration of antimicrobial agents into the matrix. Although the use of proteinase K is expensive in the poultry industry, the effectiveness of the tests opens the prospects for the chemical industry to the development of other similar proteolytics and of lower cost, since they will probably not require the necessary purity to be used in molecular techniques.

Considering the proteic nature of biofilms, it is possible that the association of potent proteolytics in association with sanitizers is an adequate strategy in the prevention of *C. jejuni* biofilms.

The centesimal composition of MH and chicken juice was compared and it was found that the analysis of 100 mL of chicken juice has 2.79% of protein and 0.06% of carbohydrates. MH contains 1.85% protein and 0.2% of carbohydrate. Even with only 5% of chicken juice in the trials, the presence of a higher protein build-up added to the existence of blood and other unassessed components may have provided *C. jejuni* not only with the microaerophilic condition required for this microorganism, as well as a greater presence of iron, important conditions for its metabolism and consequent survival and multiplication, which may have had a positive influence on biofilm formation.

For the mixed biofilms it was observed that there was an increase in the formed biomass. This increase was significant depending on the microorganism to which the interaction occurred and the medium used. In addition, there was variability in the composition of the formed biofilm.

The competitive disadvantage of *C. jejuni* visualized in the SEM indicates that probably the identified variations in biomass and in the constitution may be more related to the characteristics of the other species than to the interaction itself.

SEM images demonstrated that the configuration of mixed biofilms presented the same pattern found in the monospecific biofilm of *C. jejuni*, in both MH and chicken juice. The exception was restrict to the interaction with *P. aeruginosa* that presented in addition a more compact and flat conformation with the presence of well delimited pores, and it was also identified a higher biomass in MH in comparison with chicken juice, that presented a significant difference (*p* < 0.001) in the colorimetric assay.

The predominance of the other species in detriment of *C. jejuni*, in mixed biofilms, may be related to the biofilm formation time, since *C. jejuni* is a fastidious and demanding specie. In addition, the prevalence of other species in mixed biofilms has also been described previously and may indicate the existence of selection pressure exerted under *C. jejuni* in the first days.

According to Culotti and Packman (2015) only after 3 days of formation of the mixed biofilm of *C. jejuni* and *P. aeruginosa* was it possible to observe the presence of dispersed and discrete colonies of *C. jejuni*, which were present only on the surface of the biofilm formed by *P. aeruginosa*. In addition, the authors also detected that there was a predominance of *P. aeruginosa* biofilm morphology that remained unchanged in the *C. jejuni* presence and exhibited the same typical characteristics of the simple *P. aeruginosa* biofilm.

Several authors have already stated that both, co-inoculation and the inclusion of *C. jejuni* in pre-established biofilms facilitates subsequent growth of the sessile form of this agent (Zhang et al., 2013; Culotti and Packman, 2014, 2015).

Aswathanarayan and Vittal (2013) have suggested that different bacterial species secrete enzymes that modify the composition of extracellular polymeric substance (EPS) of biofilms in response to external stresses, resulting in changes in the biofilm architecture in a specific environment. In this way, the inclusion of different species in two substrates (MH and chicken juice) promoted these modifications.

The exception found in mixed biofilms with *P. aeruginosa* in chicken juice may represent a specific characteristic of this specie. Many animal macromolecules have been reported with the ability to form an adherent film, but not always capable of improving biofilm formation. For example, bovine serum albumin reduces formation of biofilms in *S. aureus* (Xu et al., 2008) and *Burkholderia cepacia* (Hwang et al., 2012). On the other hand, it is important for adhesion in *Cronobacter* (Healy et al., 2010). These differences also correlate with changes in the ability to express absorption proteins, leading to a variability in time to biofilm formation (Brown et al., 2015). In addition, the composition of the *P. aeruginosa* biofilm matrix is predominantly of polysaccharides, mainly alginate (Mann and Wozniak, 2012), which confers a differentiated structure, which can be detected in SEM and may represent another explanation for difficulty in adherence in the presence of chicken juice.

### Genotyping

The high heterogeneity found in *C. jejuni* strains is due to the fact that most of them are naturally competent to take the DNA present in the environment and promote recombination in their genome, that is, they execute the transformation mechanism effectively, due to production of extracellular DNAse (Clark et al., 2014).

The presence of strains with high percentage of phylogenetic similarity in different flocks and in the same one, was also reported by other authors who stated that slaughter conditions may be the main responsible for the presence of strains with a high degree of homology in samples from the same flock, such as the equipment used in animal processing and cross-contamination (Petersen and Wedderkopp, 2001; Workman et al., 2008).

Our approach has shown that the ability of *C. jejuni* in developing a structured biofilm is highly variable depending on the strain when maintained in MH. However, when there is supplementation with chicken juice, all strains present a strong biofilm producer pattern. The chicken juice allows a greater fixation of *C. jejuni* as it assigns a surface more conditioned to bacterial adhesion.

Genome analysis revealed the high potential of strains in the acquisition of sessile lifestyle, phenotypically proven in chicken juice. Its variable behavior in MH and chicken juice, apparently results from modifications in the expression of genes involved in stress response, adhesion and biofilm formation.

The existence of tolerant strains to the tested biocides and most used in the poultry industry suggests the existence of exposure to sublethal concentrations, representing a real risk for the development of adaptation mechanisms.

The ultrastructure of simple and mixed biofilms showed the early maturity range when in chicken juice compared to MH. However, in biofilms with *P. aeruginosa* this pattern is inverted, probably due to the particular characteristics of this species.

Identification of the predominantly protein composition of *C. jejuni* biomass and also in mixed biofilms may aid in the future development of agents of action with proteolytic approach as a prevention and strategy of control. However, it is noteworthy that in mixed culture with *P. aeruginosa* there is predominance of a polysaccharide matrix.

Phylogenetic diversity was evidenced by the presence of 23 pulsotypes, which confirms the intrinsic characteristic of *C. jejuni* to easily recombine its genome by gene transformation.

## Author contributions

RM: Elaborated the project, put into practice the techniques that were not yet standardized by the team and conducted the analysis of the results, application of statistics in all tests and discussion of the study. EM: Responsible for molecular analyzes, including elaboration of the PFGE dendrogram and in the preparation of samples for analysis in SEM. GM: Manipulation of *Campylobacter* strains for biofilm formation and inhibition tests, including control of the initial inoculum. MS: Performed the replicates of the tests involving the quantification and classification of *C. jejuni* biofilms. CP: Performed the replicates of the biofilm inhibition tests. PP: Molecular analyzes to evaluate the presence of specific genes. HF: Helped in the discussions of the work and in the inclusion of new ideas. DR: Guidance in the writing of the results and discussion and correction of the final paper.

### Conflict of interest statement

The authors declare that the research was conducted in the absence of any commercial or financial relationships that could be construed as a potential conflict of interest.
